# Structures of multivariables affecting literacy: Cluster analysis

**DOI:** 10.1371/journal.pone.0310114

**Published:** 2024-10-29

**Authors:** Remziye Akarsu, Gonca Bumin, Yusuf Celik

**Affiliations:** 1 Deparment of Occupational Therapy, Faculty of Health Sciences, Biruni University, İstanbul, Turkey; 2 Deparment of Occupational Therapy, Faculty of Health Sciences, Hacettepe University Ankara, Ankara, Turkey; 3 Department of Biostatistics, Faculty of Medicine, Biruni University, Istanbul, Turkey; The University of Jordan, JORDAN

## Abstract

Early literacy skills, the foundational abilities necessary for the development of literacy, must be examined holistically in preschool children. This study aimed to investigate early literacy skills in preschool children and determine how variables related to literacy development can be clustered by using a dendrogram. The study was conducted on 166 preschool children (75 female, 91 male; mean age: 65.9±4.4 months). Hierarchical cluster analysis (CA) was used to find the variable clustering trends. The 44 variables related to literacy (sociodemographic factors related to the child and family, child–parent relationship, child’s behavior, and social, sensory integration, motor, and auditory linguistic skills) that were closest to each other in the dendrogram were clustered, and the situation was summarized by reducing them to two main clusters and six sub-clusters. This study elaborates on the implications of reducing multivariate complexity using CA. It is recommended that the relationships among the variables in this dendrogram structure are considered when new hypotheses and studies related to early literacy are designed.

## Introduction

Literacy is the ability to read, write, speak, and listen in a way that allows us to communicate effectively and understand the world [1]. Different researchers use different terms to describe the prerequisites for the development of literacy skills, such as early literacy skills [2], emergent literacy skills [3], or preliteracy skills [4]. Early literacy is all of the prerequisite knowledge, skills, and attitudes that individuals are expected to acquire before they start formal literacy education [5].

Various early literacy skills exist. From the process-oriented perspective, these prerequisites are related to multiple foundational abilities in, for example, sensory integration and processing functions, the regulation of arousal level and attention control, and visual perceptual, motor, auditory linguistic, and executive functions [6–12].

The organization of sensory inputs to enable the brain to generate beneficial perceptions, emotions, and behaviors in addition to a valuable and meaningful physical response is known as sensory integration. The three separate but connected processes that comprise sensory integration are as follows: 1) sensory registration: the process by which the reception of sensory stimuli influences an individual’s alertness and ability to orient, respond, and become habituated to stimuli; 2) sensory modulation: the brain’s ability to maintain an optimal range of performance and adapt to specific life challenges by regulating and organizing the degree, intensity, and nature of responses to sensory input in a graded range of manners; and 3) sensory discrimination: the primary process of differentiating and organizing the spatial (space) and temporal (time) aspects of sensory stimuli once they are detected and modulated using intrasensory (inside a single sensory system) and intersensory (between several sensory systems) processes [13].

Poor sensory modulation has an association with a child’s behavior in the areas of arousal, attention, affect, and action [14]; leisure preferences and participation patterns [15]; adaptability, resiliency, and challenging behavior [16]; conduct, inattention, and hyperactive behavior [17]; and levels of participation in physical activity and daily occupations [18]. Children with sensory modulation problems may be hypersensitive to certain sensory stimuli or constantly seek sensory input, such as fidgeting or touching things around them. In a traditional classroom setting, these sensory issues can significantly impact their ability to focus and learn [19]. Poor sensory discrimination will affect the development of postural control, praxis, perception, and, subsequently, motor skills and behavioral organization, which are essential for different learning processes, including the development of literacy skills. Sensory discrimination skill contributes to sensory integration by enabling individuals to perceive, process, and interpret sensory information from the environment. The literature mentions the relationship between discriminating tactile, proprioceptive, vestibular, visual, and auditory sensory information and the development of literacy skills. For example, visual discrimination skills assist readers in distinguishing between letters and words on a page, while auditory discrimination skills enable them to understand the sounds represented by these symbols [6].

The complex integration of sensory modalities is required for visual perception [20]. Moreover, it has been well established in some studies that visual perception and visual motor integration are among the essential foundation abilities necessary for developing reading and handwriting skills [7, 8]. Visual perceptual and visual motor integration functions enable a child to recognize and differentiate different forms of numbers and letters, learn the correct directionality and spacing in forming letters and words, match the phoneme to the grapheme of a letter, develop consistency and persistency in handwriting performance, contribute to the development of automaticity in handwriting, and facilitate the acquisition of the fundamental processes in reading skills [21–26].

In addition to visual perceptual and visual motor functions, motor functions are fundamental to literacy development. Motor skills enable a child to develop upright and good sitting posture, shoulder stability, dissociation of arm movement, appropriate forearm and wrist posture, and dynamic control of a writing instrument by coordinating all the extrinsic and intrinsic muscles within the hand. Actions and cognitions during handwriting, which establish sensory–motor memory traces, can facilitate the acquisition of written language in reading and spelling tasks [27]. Failure to gain the necessary motor skills can have a negative impact on writing fluency and quality [28].

One of the factors whose importance in the development of literacy is frequently mentioned in the literature is auditory linguistic skills. This category consists of sub-skills such as vocabulary knowledge, phonological awareness, print awareness, letter knowledge, and listening comprehension [10, 29, 30]. Auditory linguistic functions refer to the capacity of individuals to understand and express themselves in both written and oral forms. Writing development involves activating intentions, translating ideas into grammatical strings of words, semantic retrieval, syntax, spelling patterns, selection of allographs, and strength in oral language skills. Auditory linguistic processes are required to generate text at the word and sentence level [31, 32]. Strong auditory linguistic skills form the foundation of learning across all curriculum areas. A well-developed knowledge of oral language usage allows children to manage and direct their activities as learners and to successfully engage in classroom environments where the abstract use of language occurs. Auditory linguistic skills are thus a cornerstone of literacy development [33].

In addition to all these factors, a child’s self-regulation will contribute to the literacy process. Self-regulation skills, which are necessary for the child to regulate their behavior and adapt to the environment, contribute significantly to school readiness. Additionally, there are strong connections between behavioral regulation, executive functions, and the development of literacy skills [11, 12, 34–36]. Executive functions are the cognitive skills that regulate thought, emotions, and behavior and include inhibitory control, working memory, and cognitive flexibility. These skills are crucial to various aspects of life, from paying attention to problem-solving to social interactions to academic success [37]. Among kindergarten-age children, positive correlations are found between teacher-rated self-regulation and directly measured executive function. The correlation between executive function skills and engagement in literacy-related tasks in the classroom has been highlighted. Together, these results show how crucial it is to consider executive function and behavioral regulation when analyzing how children acquire reading skills in various settings [38, 39].

Various environmental factors can also contribute to or affect the child’s acquisition of literacy skills, for example, an appropriate learning environment, parental support, parent–child interaction, and cultural influence. The home learning environment encompasses what parents do to encourage children’s literacy and mathematical skills as well as their general beliefs and attitudes about children’s learning. Parents significantly affect the development of their children’s literacy skills before children start formal education, and they play a critical role in developing opportunities for early learning [40]. In different geographical locations, the socio-cultural context plays a crucial role in the educational experiences parents provide for their children at home. It is stated that cultural values and social factors are essential in shaping parenting styles and the learning environment at home and, accordingly, impact early literacy development [41–43].

A review of the literature clearly reveals that literacy is not dependent on a single dimension; rather, it is affected by many factors, such as sensory integration, visual perception, and motor, auditory linguistic, self-regulatory, and environmental factors [6, 7, 9, 23, 36, 44, 45]. Multivariate statistical techniques examine all factors collectively to produce more accurate results. The advantage of these methods is that they take all the variables together and thus prevent information loss to give more reliable and sensitive results. One such data analysis method, hierarchical clustering analysis (CA), arranges data points into clusters or groups according to a set of shared traits. A dendrogram, a tree-like diagram that records the sequences of merges or splits, can visualize a hierarchical CA [46]. In this context, the child’s skills affecting the reading–writing process were examined holistically, and the most interrelated variables were clustered and visualized through a dendrogram. The purpose of this study is to investigate early literacy skills (sociodemographic factors related to the child and family, child–parent relationship, child’s behavior, and social, sensory integration, motor, and auditory language skills) in preschool children and determine variable clustering trends using a dendrogram. These trends provide a better understanding of which variables are strongly related to each other and show that the solid or weak skills identified in children may be linked to any skill in the same cluster. In this way, all educators, clinicians, and trainers interested in early literacy can plan for a goal-oriented approach by evaluating the variables in the same cluster together.

## Materials and methods

This cross-sectional study investigates the factors affecting preschool children’s early literacy skills.

The study was approved by the Clinical Research Ethics Committee of Biruni University on 18.11.2021 (decision number 2021/61-35). Written informed consent was obtained from the parents before the study began, and all the children gave their verbal consent to participate.

### Participants

One hundred and seventy children from six different kindergartens in different districts of Istanbul participated in this study. The participants were studying in private kindergartens, specifically in KG2 level. The inclusion criteria for the study were to be between 60 and 72 months old, attend a kindergarten, and not know how to read and write. Children with neurological, orthopedic, or psychiatric diseases may have severe delays in some of the skill areas evaluated. Additionally, due to the nature of the assessment tools used in the study, different standards may need to be applied in the data collection process for visually and hearing-impaired children. Hence, children diagnosed with any neurological, orthopedic, or psychiatric disease and with visual and hearing impairments that would affect the evaluations were excluded from the study. The children were not diagnosed with language or academic problems. Following the literature, taking the expected rate as 50% and according to 80% power, it was calculated in the R (software/ programming version 3.6.2 –CRAN) program that at least 155 children should be recruited [47]. After meetings were held with the kindergarten administrators to provide them with information, a list of contact numbers of the registered children who met the inclusion criteria was sent to the researchers. Of the parents contacted by telephone to be given information about the study, 170 volunteered to participate. Evaluations thus started with 170 children and their parents. One participant was subsequently excluded because they did not understand the task instructions, and three further participants were excluded because of incomplete assessments.

Therefore, all analyses were performed on 166 typically developing preschool children (75 female, 91 male; mean age: 65.9±4.4 months). Coincidentally, most of the participants were boys in the kindergartens where the research took place. Therefore, the gender distribution was not homogeneous. Although it was not among our criteria for the participants to be of middle socioeconomic status (SES), the kindergartens that agreed to participate in the study were in the middle socioeconomic environment. Kindergarten administrators also confirmed that the participants were of middle SES; hence, the study can be generalized to the middle-SES group. Most of the parents were high school or university graduates. The participants consisted of children whose native language was Turkish, according to the parents’ reports.

### Procedure

Children were called according to the order on their class lists and evaluated in a quiet room inside the school. The researchers (Therapist RA and Therapist GB) administered the Test of Early Literacy (TEL) [48], the Bruininks–Oseretsky Test of Motor Proficiency- the Second Edition–Brief Form (BOT-2-BF) [49], and the Motor Free Visual Perception Test–3 (MVPT-3) [50] in three 30-minute sessions between the beginning and end of the second semester of the academic year (15.02.2022 and 31.05.2022).

The researchers held a face-to-face meeting in the kindergarten to present the Family Child Information Form, Sensory Profile [51], and Child–Parent Relationship Scale [52] to the parents of the children. These assessment tools, which were filled out by parents during the meeting, collect sociodemographic information about the child and family, the child’s sensory profile, and the relationship between the child and their parents. During the meeting, the researchers checked whether the parents correctly understood the questions on the Family–Child Information Form. The other forms are standard scales adapted to Turkish that the parents could fill out independently. The parents were reminded to complete every item using the evaluation tools. At the end of the meeting, the parents filled out the forms and handed them over to the researchers. The children’s teachers were informed about the Child Behavior Rating Scale (CBRS) [53] and reminded that the form should be filled out separately for each child and that the information provided should reflect the child’s current situation. The forms filled out by the teachers for each child separately were delivered to the researchers. Confidentiality was ensured since the research participants, being preschool children, were a sensitive group, and possible conflicts of interest were prevented. In this context, ethical standards were followed during data collection, storage, and analysis.

The collected evaluation forms were stored in files created by the researchers for each child. The primary researcher assumed responsibility for the storage and preservation of the data obtained during the data collection process. The stored data were coded as P1 (Participant 1), P2, P3 … according to the order of the interviewed participants. This system ensured that there was no confusion in the data. Except for the Family–Child Information Form used in the research, the data collection tools did not include the participant’s name/surname and contact information, only the name/surname initials. Name/surname and contact numbers were not used so that third parties could not identify the participants, and they were not shared with other people. The information obtained from all data collection tools that did not contain personal data was stored on the primary researcher’s computer and SSD hard disk.

### Measures

Turkish researchers validated all the measures used.

### The family–child information form

The parents filled out the form created by the researchers to obtain sociodemographic information about the family and the child. The Family–Child Information Form contains the following information: the child’s age, gender, number of siblings, order of birth, education duration in kindergarten, parents’ age, parents’ highest level of education, child’s bedtime, parents’ reading habits, reading a book to the child, frequency of reading to the child, child’s daily TV watching time, and child’s daily internet time.

### The sensory profile

The Sensory Profile, created by Winnie Dunn in 1999, is a tool used to assess the sensory behaviors of children aged three to 10. The survey, which parents complete, consists of 125 questions categorized into three sections: sensory processing (related to different sensory systems), modulation, and behavior and emotional responses. Upon completion of the survey, nine-factor scores and three-section scores are generated. Each question is rated on a scale from 1 to 5 (1 = always, 5 = never) [54]. The test’s validity and reliability in the Turkish language were established by Kayıhan et al. in 2015. To test the validity and reliability of the study, 144 children with autism and 101 typically developing children between the ages of three and 10 were included (Cronbach’s alpha: 0.63–0.97; intraclass correlation coefficient (ICC)>.90). The results are interpreted as “typical performance,” “probable difference,” or “definite difference” under the headings “less than others” or “more than others.” A lower score indicates lower performance [51]. In this study, three-section scores (sensory processing, modulation, and behavior and emotional responses) and nine-factor scores (sensory seeking, emotionally reactive, low endurance/tone, oral sensory sensitivity, inattention/distractibility, poor registration, vestibular sensitivity, sedentary, fine motor/perceptual) were computed and utilized to evaluate children’s sensory integration skills [51, 54].

### The bruininks–Oseretsky test of motor proficiency–the second edition–brief form

The BOT was first published in 1978 by Robert Bruininks. The initial edition included a long form with 46 tasks and a short form with 14 items in one combined battery of 48 items [55]. In 2005, the test was updated and republished as BOT-2, which provides a comprehensive assessment of gross and fine motor skills in individuals aged four to 21. BOT-2 offers various options for administration, including a short form for screening, a fine motor form, a gross motor form, and the complete form [56]. The BOT-2-BF, published in 2010 by Bruininks and Bruininks, comprises eight subtests that evaluate fine motor precision, integration, manual dexterity, bilateral coordination, balance, speed and agility, upper-limb coordination, and strength. The test typically takes 15–20 minutes to administer, and the highest possible score one can achieve is 72 points [57]. Turkish researchers conducted a validity and reliability study on the BOT-2-BF, which resulted in a Cronbach’s alpha coefficient of 0.78 for the test. The study also found moderately reliable subtests, such as the BOT-2-BF Fine Motor Precision Subtest (ICC = 0.57) and Manual Dexterity Subtest (ICC = 0.74), while the remaining subtests were determined to be highly reliable (ICC>0.8) [49]. In this study, we used this tool to evaluate children’s fine and gross motor skills performances.

### The test of early literacy

The TEL was designed to assess the auditory linguistic skills of 60–72-month-old Turkish children attending preschool. It measures seven sub-dimensions: receptive vocabulary, expressive vocabulary, functional knowledge, general naming, letter knowledge, phonological awareness, and listening comprehension. The test consists of 102 items; correct answers are scored 1, while incorrect answers are scored 0. The study findings indicate that the seven-factor structure of TEL is a valid model, making it a reliable test for assessing auditory linguistic skills in 60–72-month-old Turkish children. The reliability coefficient Cronbach’s alpha for the 18 sub-dimensions was calculated as 0.85 [48]. We used TEL to evaluate auditory linguistic skills, which are significant for early literacy, through individual performance-based assessments of the participating children.

### The motor free visual perception test–3

Colarusso and Hammill (2003) developed the MVPT-3, which assesses the visual perception abilities of individuals between the ages of four and 94 without regard to their motor capabilities. About 20 to 30 minutes are needed to complete the test. On a white background and within specific guidelines, the test is normative. The test taker is presented with specific visual information and asked to select the correct response from a list of possibilities. The MVPT-3 test comprises 65 items measuring visual discrimination, shape formation, visual memory-I, visual proximity-I, visual discrimination, position in space, figure-ground, visual proximity II, and visual memory II. The first 40 items of the test are administered to people under the age of 10. Correct answers are scored 1, while incorrect ones score 0. The final score depends on the number of accurate responses. The MVPT-3 is designed to provide a general visual perceptual score. It yields a single raw score, which can be converted to a standard score, percentile rank, and age equivalent. The test manual has a table that can convert the standard score to other derived scores, for example, scaled scores, T-scores, and stanines [58]. Metin and Aral evaluated the MVPT-3 test’s validity and reliability for the Turkish language (Cronbach’s alpha: 0.85) [50]. In this study, we assessed the participating children’s visual perception skills by converting their total score from MVPT-3 into a standard score.

### The child behavior rating scale

The CBRS is an observational tool the teacher uses to evaluate children’s behavioral regulation and social skills in the classroom. The test was prepared to assess the self-regulation abilities of children aged between three and six. Children’s behaviors such as making plans, completing a given task or getting work started, cooperating, working with their friends, being organized, and exhibiting environmentally appropriate behavior are evaluated by their teachers, who are in constant interaction with the children in their classroom. The CBRS is evaluated by scoring according to the frequency of behaviors (1–never, 2–rarely, 3–sometimes, 4–often/mostly, 5–always). In the internal consistency reliability analysis performed on the 17-item scale, the Cronbach’s alpha coefficient of the items was found to be .89 and .95. The test–retest reliability of the assessment tool was found to be .67 [59]. The scale’s Turkish validity and reliability study was conducted. In the survey conducted by Sezgin and Demiriz, data were taken from 591 children between the ages of 36 and 72 months attending a preschool institution. The Cronbach’s alpha reliability coefficient for the scale’s sub-dimensions was between 0.84 and 0.96. The research demonstrated that the Turkish form of the scale is a valid and reliable tool for measuring preschool children’s self-regulation skills [53]. In this study, we used CBRS, an observation-based assessment tool, for teachers to score children’s behavioral regulation and social skills in the classroom.

### The child–parent relationship scale

Robert C. Pianta’s scale, developed in 1992, has been instrumental in understanding the parent–child relationship [60]. The original scale, with its three sub-dimensions and 30 items, served as the foundation for the Turkish version, which consists of 24 items and two sub-dimensions: conflict and positive relationship. A validity and reliability study of the scale for children aged four to six was conducted in Turkey. The scale, with its inclusion of positive and negative expressions, is scored in reverse order for the negative expressions and is structured around conflict and positive relationships. The test–retest reliability coefficients are .97 for the conflict sub-dimension, .87 for the positive relationship sub-dimension, and .95 for the total score. The internal consistency coefficients (Cronbach’s alpha) are .70 for the overall scale, .85 for the conflict dimension subscale, and .74 for the positive dimension subscale. A high score indicates a negative relationship, while a low score indicates a positive relationship. The total score ranges between the highest (75; most negative) and the lowest (15; most positive) [52]. In the study, we requested that parents fill out this scale to understand the relationship between each child and their parents.

### Data analysis

The Kolmogorov–Smirnov test was used to assess data normality [61]. As a result, most data were analyzed using non-parametric statistical methods. Median and interquartile ranges were used to describe ordinal data, and mean and standard deviations were used to measure continuous variables.

The clustering pattern of the variables (age, education duration in kindergarten, mother’s age, father’s age, gender, number of siblings, order of birth, mother’s highest level of education, father’s highest level of education, child’s bedtime, parents’ reading habits, reading a book to the child, frequency of reading to the child, child’s daily tv watching time, child’s daily internet time, semantic knowledge, phonological awareness, letter knowledge, listening comprehension, visual perception, fine motor precision, fine motor integration, bilateral coordination, upper-limb coordination, speed and agility, manual dexterity, balance, strength, child–parent positive relationship, child parent conflict, behavioral regulation, social skills, behavioral and emotional responses, emotionally reactive, poor registration, sensory processing, sensory seeking, modulation, inattention/distractibility, low endurance tone, vestibular sensitivity, sedentary, fine motor/perceptual, and oral sensory sensitivity) was discovered using hierarchical CA, a contemporary multivariate statistical technique. The process of hierarchical CA involves grouping similar objects into clusters based on their similarities. The goal is to have similar objects within a cluster while keeping separate clusters distinct. To illustrate this process, a dendrogram is created using either a top-down (divisive) or bottom-up (agglomerative) approach. A dendrogram, which visually connects things that appear to be related, was used to illustrate the relationships between the variables. The common linkage (between groups) and Ward’s hierarchical clustering model were used to find the dendrogram of the variables. By combining more than one variable, hierarchical CA can study interacting relationships between variables. To examine interaction outcomes and create clusters to identify the connections between variables or to establish the circumstances under which the association occurs, hierarchical CA can govern the association between variables. Compared to using a single variable, this produces a richer and more accurate picture and offers a strong test of significance. As a result, the strategy provides better outcomes than univariate methods so that the researcher may have a more substantial research thesis [46].

## Results

The research was conducted on 166 typical-developing preschool children (75 female, 91 male; mean age: 65.9±4.4 months). Tables 1 and 2 present sociodemographic descriptive information about the children.

**Table 1:** Descriptive statistics- frequency distributions of children and families.

NN:166 = 166 n	N (%)N (
**Gender**	
• Female	75 (45.2)
• Male	91 (54.8)
**Mother’s Highest Level of Education**	
• No literacy	6 (3.6)
• Primary school	28 (16.9)
• High school	36 (21.7)
• University	86 (51.8)
• Master Degree	10 (6)
**Father’s Highest Level of Education**	
• No literacy	2 (1.2)
• Primary school	30 (18.1)
• High school	56 (33.7)
• University	72 (43.4)
• Master Degree	6 (3.6)
**Child’s Bedtime**	
• 19.00–20.00	6 (3.6)
• 20.00–21.00	6 (3.6)
• 21.00–22.00	76 (45.8)
• 22.00 and above	78 (47)
**Parent’s Reading Habits**	
• Read	104 (62.7)
• Rarely read	46 (27.7)
• Not read	16 (9.6)
**Reading a Book to Child**	
• Read	146 (88)
• Not read	20 (12)
**Frequency of Reading to the Child**	
• Daily	64 (41.5)
• Once in 2–3 days	48 (31.2)
• Once a week	28 (18.2)
• Once a month	14 (9.1)

**Table 2:** Descriptive statistics- mean and median values of children and families.

NN:166 = 166 n	Mean (SD)	Median (IQR)
**Age (months)**	65.9 (4.4)	
**Kindergarten starting age (years)**	4.3 (0.9)	
**Education duration in kindergarten (years)**	1.5 (1.3)	
**Mother’s age (years)**	35.3 (4.7)	
**Father’s age (years)**	38.7 (5.1)	
**Child’s daily TV watching time (hours)**	2.2 (1.9)	
**Child’s daily internet time (hours)**	1.3 (1.3)	
**Number of siblings (n)**		1 (0–2)
**Order of birth (n)**		1 (1–2)

SD = Standard Deviation; IQR = Interquartile Range

TEL, MVPT-3, CPRS, BOT-2-BF, CBRS, and Sensory Profile standard scores were presented in Table 3.

**Table 3:** Descriptive statistics-children outcomes.

NN:166 = 166 n	Median (IQR)
**Test of Early Literacy**	
• Semantic Knowledge	32 (26–37)
• Phonological Awareness	13 (11–17)
• Letter Knowledge	3 (1–6)
• Listening Comprehension	3 (2–4)
**Motor-Free Visual Perception Test 3**	66 (55–90)
**Child-Parent Relationship Scale (total)**	
• Conflict	31 (26–38)
• Positive Relationship	19 (18–22)
**Bruininks-Oseretsky Test of Motor Proficiency-2-Brief Form**	
• Fine Motor Precision	6 (4–7)
• Fine Motor Integration	7 (5–8)
• Manuel Dexterity	3 (2–4)
• Bilateral Coordination	4 (3–6)
• Balance	3 (2–4)
• Speed and Agility	4 (2–7)
• Upper-Limb Coordination	0 (0–2)
• Strength	2 (0–3)
**Child Behavior Rating Scale**	
• Behavioral Regulation	46 (37–49)
• Social Skills	30 (28–33)
**Sensory Profile**	
• Sensory Processing	274 (257–294)
• Modulation	141 (128–156)
• Behavioral and Emotional Responses	102 (90–110)
• Sensory Seeking	67 (60–77)
• Emotionally Reactive	61 (51–69)
• Low Endurance/Tone	40 (35–44)
• Oral Sensory Sensitivity	34 (27–40)
• Inattention/Distractibility	29 (26–33)
• Poor Registration	37 (33–39)
• Vestibular Sensitivity	19 (16–20)
• Sedentary	18 (14–19)
• Fine Motor/Perceptual	12 (10–13)

IQR = Interquartile Range

### Clusters

**Fig 1:**
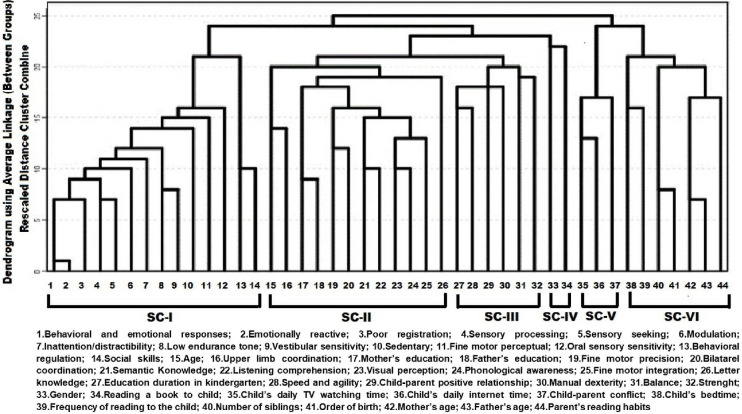
Dendrogram.

Hierarchical CA uses all variations to find close relationships between relevant variables. This technique reveals the relationships between variables more clearly than other methods, as using a dendrogram prevents the loss of information. The analysis used a Z score for data transformation to determine the main clusters and sub-clusters (SCs). The clustering method simplifies the relationships between variables, leading to more precise results. Each SC contains the variables most strongly linked to each other, which are visually closer in the dendrogram structure [46]. At the end of the research, two main clusters and six SCs were obtained using a dendrogram (Fig 1).

The main cluster-I (MC-I) contains four SCs. Each SC structure was named based on the characteristics of the variables within it (Table 4).

**Table 4:** Variables of main-clusters and subclusters.

**MAIN-CLUSTER I**				**MAIN CLUSTER-II**	
**Subcluster-I**	**Subcluster-2**	**Subcluster-III**	**Subcluster-IV**	**Subcluster-V**	**Subcluster-VI**
Sensory Integration and Self-Regulation	Linguistic, visual, fine motor skills, and sociodemographic characteristics	Preschool education, child-parent, and gross motor skills	Gender and reading habits	Technology use and parent-child conflict	Home literacy environment, routines, family structure
Behavioral and emotional responses Emotionally reactive Poor registration Sensory processing Sensory seeking Inattention/distractibility Modulation Vestibular sensitivity Sedentary Fine motor/perceptual Oral sensory sensitivity Behavioral regulation Social skills Low endurance tone	Age Upper limb coordination Mother’s highest level of education Father’s highest level of education Bilateral coordination Semantic knowledge Listening comprehension Visual perception Phonological awareness Fine motor integration Letter knowledge Fine motor precision	Education duration in kindergarten Speed and agility Child-parent positive relationship Manual dexterity Balance Strength	Gender Reading a book to the child	Child’s daily internet time Child’s daily TV-watching time Child-parent conflict	Child’s bedtime Frequency of reading to the child Number of siblings Order of birth Mother’s Age Father’s Age Parent’s reading habits

## Discussion

To the best of the authors’ knowledge, having reviewed the literature, this study is the first to comprehensively address early literacy skills in preschool children and present inclusive results using a hierarchical CA. A dendrogram has reduced multiple variables in the complex structure researchers have examined related to literacy, creating a new visual structure. Considering this structure, which interprets the relationships between variables (sociodemographic factors related to the child and family, child–parent relationship, and child’s behavioral, social, sensory, motor, and auditory linguistic skills), will provide highly beneficial information for those formulating new hypotheses and planning research related to literacy.

The variables are gathered under two main clusters. There are four SCs under Main Cluster-1 (MC-1) and two under Main Cluster-2 (MC-2). The cluster method is a multivariate statistical technique that identifies grouping patterns for variables in the form of a dendrogram and generates new visual clusters. Each cluster brings together variables that have a high level of relationship. The hierarchical positioning of a variable at a higher level implies it significantly influences the variables within its cluster. Whichever variable stands higher in each SC has a stronger relationship with the other variables. The variables that form an SC structure are closer to each other, indicating a higher level of relationship among the variables. As a result, the variables covered by each cluster present the relationships that should be primarily considered regarding the topic. The decision-making process can benefit from statistical CA [46].

MC-I contains four SCs. SC-I includes variables related to the child’s sensory profile, behavioral regulation, and social skills. Evidence suggests that self-regulation abilities govern many characteristics and behaviors associated with cognitive school readiness, and higher self-regulation is linked to better school readiness outcomes [62]. Integrated behavioral regulation skills are necessary to complete the learning process successfully. Studies indicate that behavioral regulation skills strongly predict literacy, such as reading comprehension, accuracy, and fluency [63]. In early education, behavioral regulation is associated with academic and social performance [11]. Due to the nature of education, children must constantly utilize executive and self-regulation skills. For instance, students must adhere to instructions, follow classroom guidelines, solve mathematical problems, comprehend texts, cooperate with peers and teachers, recall equipment, and apply information across various scenarios [39].

A strong relationship between the child’s sensory profile, behavioral regulation, and social skills is observed within the SC-I structure. The body constantly receives sensory information, including through vision, hearing, smell, taste, touch, movement, and proprioception, from the environment and different parts of the body. The brain organizes processes and integrates these sensory inputs from the sensory systems to complete daily tasks [13]. Sensory integration is essential in literacy development, increasing engagement and success in reading and writing tasks [6]. This integration allows for the planning and execution of appropriate responses to situational demands. Sensory integration is crucial for self-regulation, executive functions, and all other cognitive abilities [64]. Therefore, having well-developed sensory modulation functions is an essential prerequisite for a child’s literacy skill development, enabling their participation in various learning activities. This fundamental function should be considered alongside self-regulation skills in clinical research and educational settings. Understanding sensory systems and processing is crucial to developing enriched multisensory environments that promote learning for all students, especially those with sensory integration disorders. Children with sensory processing disorders, whether they are hypersensitive or hyposensitive, benefit from personalized measures such as adjusting teaching materials, breaking tasks into smaller units, and emphasizing key points to improve reading and writing skills [65].

Children must be able to engage in social interactions, regulate their emotions, and communicate effectively to acquire the foundational performance skills necessary for adulthood. These skills should be developed in the classroom and the play environment. Social interactions provide language exposure, opportunities for vocabulary acquisition, and comprehension practice. Peer interactions also promote collaborative learning experiences, where children engage in discussions, share ideas, and provide feedback, all of which enhance literacy skills [66, 67]. Challenges in school life may arise from the child’s inability to assimilate sensory information or develop self-regulatory responses. A child who does not have good sensory integration skills will have more difficulty entering and adapting to social environments. For example, a child who is sensitive to noise may have difficulty interacting with classmates in busy social environments [67]. Therefore, occupational therapists must understand how sensory skills and self-regulation influence a child’s participation in activities. This knowledge can promote more suitable environments for children’s literacy performance, learning, and school needs [68]. This relationship observed in SC-I can offer a different perspective on social skills, which is one of children’s learning conditions. Interpreting these skills through sensory profiles and behavioral regulation can enable appropriate guidance in educational settings.

SC-II includes age, parental education level, visual perception, fine motor skills, bilateral and upper extremity coordination, and auditory linguistic skills. Studies have reported on the impact of family factors on skills that serve as prerequisites for literacy. The home literacy environment’s quality is associated with maternal education [69] and parental SES as indicated by parental occupation [70]. The regularity of parental engagement in early literacy activities is predicted to positively impact children’s future mathematics and reading skills, as predicted by a conceptualization of child development. A child’s early literacy development can occur within the context of the environment prepared by parents, which can vary based on the parents’ level of knowledge and skills [71]. Parents’ behaviors related to literacy are associated with the entirety of literacy resources in families and children’s language skills [72]. It has been shown that visual and speech–language neural systems are related to individual differences in cognitive linguistic preliteracy skills [73]. There are findings that white matter development, which occurs with parental language input in the first two years of life and is observed at the beginning of the third year (26 months), is effective in auditory linguistic skills such as letter identification and letter–sound knowledge. Language experiences in the first two years are essential in predicting the literacy skills that emerge at the age of five [74].

Visual perception and fine motor skills, which also fall within SC-II, have often been included in preschool interventions, and their effects on school readiness have been demonstrated. Visual perception skills are crucial in the fundamental processes of reading and writing. These skills are foundational for school readiness, enabling children to recognize and distinguish various letter and number forms and to learn the correct direction and spacing when writing letters and words [23–25]. Additionally, visual perception is essential for in-hand manipulation skills and writing [25]. Successful participation in the educational process for children relies on their ability to perform fine motor tasks [75]. Kindergarten readiness is linked to parental knowledge about child development and the development of fine motor skills [76, 77]. According to research, the capacity to perform daily tasks related to personal care and academic activities in kindergarten significantly correlates with fine motor skills [78]. Cameron et al. (2016) found that American children with advanced fine motor skills at five performed better in arithmetic and reading when tested six, eight, and 10 years later [79]. Motor functions, particularly transcription skills in handwriting, are essential early literacy skills [27]. A child with coordination or motor speed problems may struggle to keep up with their peers in the classroom. These children may need more time to complete assignments in class, and it may take them longer to learn new skills [80]. The current study predicts strong relationships among language, visual, fine motor skills, coordination, and parental characteristics, similar to the literature. Addressing these skills, which are prerequisites for literacy under SC-II, is essential.

SC-III includes the duration of education in kindergarten, the positive relationship between the child and parent, and gross motor skills. A positive relationship between the child and parent is essential for literacy skills. It is emphasized that parents’ participation in reading activities, such as reading together, telling stories, and discussing texts, significantly affects the child’s reading success [81]. Additionally, studies show that parent–child interactions during shared literacy activities, characterized by parental responsiveness, positive regard, and child participation, mediate the relationship between these activities and children’s emergent literacy and oral language skills. This highlights the importance of engaging in reading activities and developing a supportive and nurturing relationship between parents and children to impact literacy development positively. Parenting styles significantly affect children’s early academic success [41, 82]. The impact of the child’s communication environment on skill development is evident; in this regard, parents have a critical role to play [83].

The current study highlights the strong relationship between the duration of education and motor skills. Evidence has shown that children spend much time at home outside school hours [84] and that much of children’s sedentary time occurs at home [85, 86]. Increasing a child’s preschool education duration can significantly enhance motor skills by limiting the time spent at home. This relationship in SC-III emphasizes that educational settings can positively support motor skills, which are one of the prerequisites for literacy.

SC-IV highlights the strong links between child gender and parents’ reading to their children. Findings indicate that book reading rates are higher for girls. It has been noted that girls outperform boys in reading when they enter kindergarten and learn marginally more throughout the academic year [87]. Evidence suggests that the quality of early home literacy environments significantly influences the diversity in early language and preliteracy skills [70, 88]. Similarly, some studies have shown gender differences in literacy environments at home, with a preference for girls [89, 90]. One study indicated that the home literacy environment provided to boys is generally poorer than that provided to girls [69]. Encouraging parent–child shared book reading in early childhood is vital to evidence-based recommendations for promoting literacy development [91, 92]. The current study reports similar results to the literature, emphasizing the importance of fostering interactive book reading in early childhood regardless of gender.

SC-V demonstrates strong links between child–parent conflict and screen time. Research has reported a significant relationship between screen use and cognitive developmental outcomes in young children, such as short-term memory skills, academic achievement, and language development [93, 94]. Increased video game play has also impacted children’s development by reducing the quality of interactions between parents and children [95]. From a theoretical perspective, social interaction theory assumes that children and parents mediate child–parent relationships [96]. Parental conflict in families with preschool children negatively affects consistent daily routines [97]. Consistent and predictable routines are essential, as they are positively associated with child self-regulation and the parent–child relationship [77, 97]. The prominent relationship highlighted in the current study is vital to demonstrating parent–child interaction within an early literacy environment. Addressing these variables in SC-V together can be critical for fostering literacy development.

SC-VI includes variables such as the child’s bedtime, frequency of reading to the child, number of siblings, birth order, mother’s age, father’s age, and parents’ reading habits. This SC structure reveals strong relationships in routines such as bedtime, parents’ reading habits, and frequency of reading to the child. Consistent family routines, book reading, and parental knowledge about child development have been associated with school readiness [76, 77, 79]. In addition to helping children sleep better, the bedtime routine can benefit language development, literacy, child emotional and behavioral regulation, parent–child connection, and family functioning [98].

The current study demonstrates the strong relationships between the presence of siblings and the increased frequency of parents reading to the child. A sibling attending school is associated with literacy resources in the home [72]. Findings also suggest that regardless of SES, children with more siblings show weaker reading and math skills than children from single-child families at kindergarten entry [99]. The relationships among these variables in SC-VI may need further investigation, excluding other factors.

### Limitations and directions for future research

The selected methodology has certain inherent limitations. Dendrograms demonstrate cluster structures based on the distance between variables. They facilitate our understanding of the correlations between variables within and between clusters but do not provide information to allow for causal inference. Consequently, future studies will profit from a more reliable design to find results other than correlations.

The study presented reviews from various sources, including direct child assessments and parent- and teacher-reported measurements. The observations of parents and teachers may not have been objective. In future studies, using standardized performance-based assessments for children could help increase objectivity.

The study’s kindergartens had more boys, which may explain the significant difference in gender distribution among the participants. Studies show that girls are better than boys in phonemic awareness, spelling, word reading accuracy, and letter identification. In contrast, boys may struggle more with writing and spelling in early childhood [100, 101]. This situation may have affected the variables of early literacy skills evaluated in the study. In future studies, we recommend that the gender distribution should be homogeneous.

The parents’ middle SES constrains the generalizability of the study’s findings. It is advised that participants from low SES be included in future studies. A literature review shows a stronger relationship between higher levels of approach to learning and better academic ability in children from lower-SES families than in diverse-SES families. Approaches to learning are a significant protective factor for the academic skill development of children from low-SES backgrounds [42]. For this reason, it would be more beneficial to consider children’s learning approaches to early literacy, especially at low SES levels.

The study did not include a specific assessment for visual–motor integration and praxis. While the BOT-2-BF used in the study was not designed to measure these skills, it did include sub-items that allowed for the observation of related skills. However, in future studies, it is recommended to use specific tools to evaluate visual–motor integration and praxis (motor planning) skills.

The initial stage of literacy skills starts when children are around four years old. For future studies, it will be appropriate to use a cohort of children aged between four and six rather than five and six.

This cross-sectional study provides information reflecting the factors affecting literacy skills in preschool children. A prospective longitudinal study following the same cohort of children from four to about seven or eight years old would be helpful for future research.

Although it has some limitations, this study extends similar studies in the literature by presenting identified factors that can be examined and targeted more concretely in interventions using a visual structure with main clusters and SCs.

## Conclusions

The visual structure presented in this study provides a comprehensive representation of the foundational abilities necessary for developing literacy. Consistent results have been obtained that are in line with the complex literature on early literacy studies, and these results are explained in a simplified framework. Variables that were close to each other in terms of distance in the dendrogram formed main clusters and SCs. Thus, the situation was summarized by reducing 44 literacy-related variables to two main clusters and six SCs. It is recommended that components more strongly related to each other be considered in clinical, educational, and academic studies on early literacy. In clinical and educational settings, when a problem with any component is detected in children, simultaneously evaluating other elements in the same SC and addressing these components together in intervention programs will significantly contribute to finding a holistic solution to the problem. The statistical method used in the study revealed the relationships clearly, as it prevented the loss of information regarding variables previously researched in the literature regarding early literacy. The relationships between variables are visualized with the dendrogram structure, and more practical and solution-related results are given. Considering that early age variables can affect children’s health and school readiness, this study’s concrete and visual structures will shed light on new interventions and research. This study provides essential information about raising awareness among professionals to consider all the child’s skills holistically in the design of education policies and early childhood intervention programs as well as about applying more practical and target-oriented approaches in disadvantaged situations. This unique structure, the first of its kind, should inspire the design of new hypotheses and studies on early literacy in the future.

## Supporting information

S1 Dataset (XLSX)
